# QuickStats

**Published:** 2015-07-03

**Authors:** FROM THE NATIONAL CENTER FOR HEALTH STATISTICS

**Figure:**
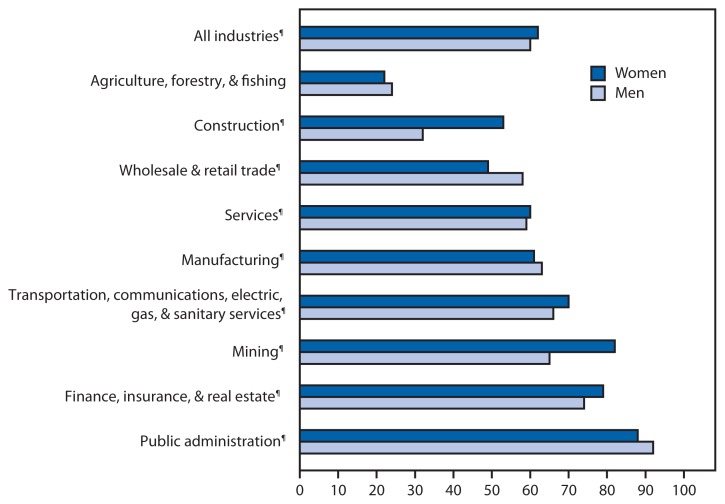
Percentage of Currently Employed Adults Who Had Paid Sick Leave,* by Industry^†^ — National Health Interview Survey, United States, 2009–2013^§^ * Based on responses to a question that asked, “Do you have paid sick leave on this MAIN job or business?” ^†^ Respondents were asked to identify the business or industry of their main job, and these industries/businesses were then categorized by the North American Industry Classification System (http://www.census.gov/eos/www/naics/). ^§^ Estimates were based on a sample of the U.S. civilian, noninstitutionalized population aged ≥18 years. Adults not currently employed at the time of interview were not included in the denominators when calculating percentages. ^¶^ The percentage difference between women and men within this category was statistically significant at p<0.01.

During 2009–2013, approximately 60% of employed men and women had paid sick leave at their main job. For both men (90%) and women (88%), paid sick leave was most common in the public administration sector and least common in the agriculture, forestry, and fishing sector (24% for men and 22% for women). Women were more likely than men to have paid sick leave in the following industries: construction; finance, insurance, and real estate; mining; services; and transportation, communications, electric, gas, and sanitary services. Men employed in the manufacturing and wholesale and retail trade industries were more likely to have paid sick leave than women in those industries.

**Source:** National Health Interview Survey, 2009–2013. Available at http://www.cdc.gov/nchs/nhis.htm.

**Reported by:** Roger R. Rosa, PhD, RRosa@cdc.gov, 202-245-0655; Abay Asfaw, PhD, Rene Pana-Cryan, PhD.

